# A role for DICAM^+^ mononuclear phagocytes in controlling neuroinflammation in multiple sclerosis

**DOI:** 10.3389/fimmu.2025.1628398

**Published:** 2025-07-28

**Authors:** Marina Rode von Essen, Marie Mathilde Hansen, Sahla El Mahdaoui, Victoria Hyslop Hvalkof, Malene Bredahl Hansen, Sophie Buhelt, Finn Sellebjerg

**Affiliations:** ^1^ Danish Multiple Sclerosis Center, Department of Neurology, Copenhagen University Hospital – Rigshospitalet, Glostrup, Denmark; ^2^ Department of Clinical Medicine, Faculty of Health and Medical Sciences, University of Copenhagen, Copenhagen, Denmark

**Keywords:** DICAM, neuroinflammation, mononuclear phagocytes, monocytes, macrophages, multiple sclerosis

## Abstract

Multiple sclerosis (MS) is a chronic inflammatory disease of the central nervous system (CNS). In MS, CNS-infiltrating monocytes differentiate to tissue resident macrophages which are found in large numbers within the injured areas of the brain where they play a central role in driving disease progression through demyelination and tissue destruction. However, infiltrating monocytes and their derivative macrophages can also serve protective functions. In this study we investigated a possible role of intrathecal mononuclear phagocytes (infiltrating monocytes and macrophages) expressing dual immunoglobulin domain-containing cell adhesion molecule (DICAM) in neuroinflammation. Compared to symptomatic controls (n = 14), treatment-naïve patients with relapsing-remitting MS (n = 21) had a reduced prevalence of DICAM^+^ mononuclear phagocytes in CSF. When patients were treated with natalizumab (n = 12), an antibody blocking migration of blood leukocytes to the CNS, we observed that DICAM^+^ monocytes were still recruited to the CSF and that the level of soluble DICAM (sDICAM) in CSF was significantly increased in natalizumab-treated patients (n = 42) compared to untreated patients (n = 43). sDICAM and the prevalence of DICAM^+^ mononuclear phagocytes in CSF furthermore correlated negatively with concentrations of various cytokines, including TNFα. Analysing the functional properties of DICAM showed that LPS-induced TNFα-production in mononuclear phagocytes was effectively reduced by signalling through surface-bound DICAM. This discovery, together with the observation of a high prevalence of infiltrating DICAM^+^ mononuclear phagocytes in individuals with no disease or in which disease was kept under control, suggests an immunomodulatory role of DICAM^+^ mononuclear phagocytes. DICAM has been shown to engage in homophilic interactions with DICAM expressed on the same cell. If sDICAM in a similar way can engage with DICAM on adjacent cells, the increased intrathecal sDICAM of natalizumab-treated patients may help regulate inflammation in a paracrine way. Overall, our data suggest that DICAM^+^ mononuclear phagocytes play a role in controlling neuroinflammation.

## Introduction

Multiple sclerosis (MS) is a chronic, immune-mediated disease of the central nervous system (CNS) with increasing incidence, affecting 2.9 million people worldwide (1). Approximately 90% are diagnosed with a relapsing-remitting form of MS (RRMS) characterized by episodic relapses of neurological symptoms. These attacks are mediated by activation of immune cells and their migration to the CNS where they participate in myelin sheath damage resulting in formation of inflammatory demyelinated lesions and neuroaxonal damage (2, 3). Microglia and macrophages are the main innate cells present in MS lesions where they directly or together with T and B cells cause neuroinflammatory tissue damage (4). It has been proposed that CNS-resident microglia contribute to clearing of debris in lesion areas and that macrophages originating from blood monocytes are drivers of immunopathology (5).

In normal, steady-state conditions, blood monocytes also infiltrate the borders of the CNS as part of immune surveillance to monitor infection, malignancy, and tissue damage. Here, infiltrating monocytes differentiate into CNS border-patrolling macrophages which only cross the blood-brain-barrier (BBB) to enter the brain parenchyma in case of damage or pathogen-associated signals (5).

With this study we investigated cerebrospinal fluid (CSF) cells of monocyte origin expressing dual immunoglobulin domain-containing cell adhesion molecule (DICAM; with the alternative names MXRA8 and limitrin). DICAM is a membrane-bound receptor with proposed BBB-migratory abilities (6), likely through its homophilic interaction with DICAM and heterophilic interaction with αVβ3 on endothelial cells (7). Also, DICAM is likely implicated in intracellular signalling pathways affecting various functions of cells including proliferation (8), angiogenesis (9), apoptosis (9) and expression of inflammatory molecules, including IL-1β, TNFα, IL-6 and integrin β1 and β3 (10). Studies have furthermore indicated an immunosuppressive role of DICAM (10–13) and implied that DICAM controls the activity of microglia (12). The aim of this study was to investigate a possible role of DICAM-expressing cells of monocyte origin during steady state in control subjects without neurological disease and in neuroinflammation in patients with RRMS. As no surface markers to clearly distinguish between a CSF-infiltrating monocyte and a differentiated border-patrolling macrophage have been described, we generally refer to CSF cells of monocyte origin as mononuclear phagocytes.

## Materials and methods

### Study population, protocol approvals and ethics

In this observational case-control study, we included CSF cell samples from 14 symptomatic control subjects, 21 treatment-naïve patients with RRMS, 12 patients treated with natalizumab, and blood cell samples from 21 healthy controls, 21 symptomatic controls, 21 treatment-naïve patients with RRMS, and 13 patients treated with natalizumab. There was no significant difference in sex or age distribution between the groups. Furthermore, cell-free CSF samples from 41 symptomatic controls, 43 treatment-naïve patients with RRMS and 42 natalizumab-treated patients were included. There was no significant difference in sex or age distribution between the groups except a higher age of natalizumab-treated patients compared to untreated patients. Symptomatic control subjects were defined as patients with symptoms warranting a lumbar puncture but with no indication of inflammation or neurological disorder after diagnostic work-up. Symptoms of the symptomatic controls included sensory symptoms, pain, tension headache, migraine, sleep symptoms (hypersomnia), or idiopathic Bell’s palsy (14). All patients were diagnosed with RRMS fulfilled the 2017 McDonald criteria (15) and none had received methylprednisolone treatment within one month prior to sampling. All treatment-naïve patients with RRMS were diagnosed at the time of lumbar puncture and had a disease duration of 10 months (3; 25); median (IQR). Patients treated with natalizumab had been treated for 38 weeks (12; 97) at sampling time, and time since last treatment was 27 days (19; 44), median (IQR). Of the 42 natalizumab-treated patients, 4 switched to another treatment at sampling time due to disease activity. Healthy controls had no neurological, autoimmune, or chronic illness.

All participants gave informed, written consent to participation. The study was approved by the regional scientific ethics committee (protocol number H-17005703 and H-16047666).

### Blood and CSF samples

Venous blood was collected, and peripheral blood mononuclear cells (PBMC) isolated by density gradient centrifugation using Lymphoprep (Axis-Shield, Oslo, Norway), washed twice in cold PBS/2 mM EDTA and applied directly to *ex vivo* flow cytometry or *in vitro* assays. In parallel with blood sampling, 10 ml of CSF was collected in a polypropylene tube on ice and immediately centrifuged for 10 min at 400 g to separate cells from fluid. Cell-free CSF was kept at -80°C until use and CSF cells applied to flow cytometry within one hour of sampling. PBMC were manually counted using a Neubauer chamber, and CSF cell counts were measured during routine assessment with counts below 3 cells/µl reported as 2 cells/µl. CSF cell count of natalizumab-treated patients below 3 cells/µl was reported as 1 cell/µl according to literature (16).

### Flow cytometry analysis

For *ex vivo* flow cytometry, freshly isolated PBMC and CSF cells were incubated in FcR-blocking reagent (Miltenyi Biotec, Bergisch Gladbach, Germany) to prevent nonspecific Ab binding, and thereafter stained in PBS/2% FBS/0.02% NaN_3_ with a combination of fluorochrome-conjugated antibodies against (conjugate; clone): CD14 (BV605; M5E2), CD16 (PerCP; 3G8), CCR2 (BV421; K036C2), CD40 (APC-Cy7; 5C3), CD49d (APC; 9F10), CD86 (PE-Cy7; 2D10), CD206 (PE; 15-2) all from BioLegend (CA, USA). DICAM (unconjugated; 2H2G12A) from MBL International Corporation (USA, IL) was conjugated using the fast AF488-conjugation kit from Abcam (Cambridge, UK), according to manufacturer. Isotype matched controls were used to correct for nonspecific Ab binding and spectral overlap, where appropriate. For *in vitro* flow cytometry a live-dead stain (Thermo Fisher Scientific, MA, USA) was included in the staining procedure. TruCount staining of whole blood to measure absolute cell count was performed using BD Multitest 6-color TBNK Reagent including a CD14 antibody (BV605; M5E2), according to manufacturer (BD Biosciences, CA, USA). Data were acquired on a FACSymphony or FACS Canto II flow cytometer (BD Biosciences) and data analysis performed using the software FlowJo (TreeStar, OR, USA).

### *In vitro* macrophage differentiation

PBMC were purified from healthy donors and monocytes negatively selected using the human monocyte enrichment kit without CD16 depletion from StemCell Technologies (Vancouver, Canada), according to manufacturer. The purity of monocytes was measured by flow cytometry of cells stained with anti-CD14 antibodies (APC-Cy7; M5E2) and live-dead stain (Thermo Fisher Scientific). Hereafter, cells were resuspended in monocyte attachment media (Sigma-Aldrich; MO, USA) at a concentration of 1×10^6^ monocytes/ml and 200.000 monocytes plated in each well of a 96 wells flat bottom plate. After 1 h at 37°C, non-adherent cells were washed off, and medium changed to 100 ng/ml M-CSF (R&D Systems; MN, USA)/macrophage base medium DXF (Sigma-Aldrich), and cells incubated at 37°C, 5% CO_2_ for 5 days. The growth medium was then replaced by fresh medium and cells incubated an additional 24 h. Hereafter, cell-free CSF was thawed and centrifuged at 3000g, 10 min to pellet any cell debris. The growth medium was removed from the cells and replaced by fresh growth medium with/without CSF from one of the 42 donors at a concentration of 37.5%. CSF was used from 14 symptomatic controls, 14 treatment-naïve patients with RRMS and 14 patients treated with natalizumab. After 48 h the cells were detached using accutase (Sigma-Aldrich) and analyzed by flow cytometry to measure expression of DICAM. For this, cells were stained with FcR-blocking reagent, DICAM-AF488 and live-dead stain as described in the *flow cytometry analysis* section above.

### *In vitro* monocyte secretion of sDICAM

Monocytes were purified as described in the *in vitro macrophage differentiation* section and plated at a concentration of 1×10^6^ monocytes/ml in RPMI with 1–1000 ng/ml LPS (InvivoGen, CA, USA). After 14 h at 37°C, 5% CO_2_ the supernatant was applied to ELISA-measurement of sDICAM (human MXRA8-ELISA; MyBioSource, CA, USA), according to manufacturer.

### TNF suppression assay

Monocytes were purified from PBMC using the human monocyte enrichment kit without CD16 depletion (StemCell Technologies), and stained with antibodies targeting DICAM (AF488; 2H2G12A) and CD14 (APC-Cy7; M5E2) in PBS/2 mM EDTA/2% FBS and 9000 CD14^+^DICAM^-^ and 9000 CD14^+^DICAM^+^ cells sorted on a BD Melody FACS at 4°C. Cells were hereafter cultured with 10 ng/ml LPS (InvivoGen) for 6h at 37°C, 5% CO_2_ and the supernatant applied to ELISA-measurement of TNFα (DuoSet from R&D Systems). The experiment was repeated using freshly drawn CSF-cells that was likewise incubated with DICAM and CD14 antibodies and 10 ng/ml LPS at 37°C, 5% CO_2_.

### Biomarker analysis

Biomarker analysis was performed on cell-free CSF samples from 41 symptomatic controls, 43 untreated patients with RRMS and 42 natalizumab-treated patients. For measurement of IL-8, IL-10, IL-12, IL-15, TNFα, and IFNγ electrochemiluminescence assays (Vplex kits) from Meso Scale Diagnostics (MD, USA) was used according to manufacturer. The maximum lower level of detection across plates measured for each analyte was used as a cut-off for detection. The limit of quantification was defined by the lowest standard with a CV below 25%, samples below this limit was assigned a value of 0. Samples were measured in duplicates and excluded if CV exceeded 20%, or 25% if the value was within the two lowest accepted standard. ELISA was used to measure CSF concentrations of sDICAM (MXRA8-ELISA from MyBioSource), NfL (Uman Diagnostics, Umeå, Sweden), and GFAP (SIMOA technology; Quanterix, MA, USA), according to manufacturer. NfL and GFAP were measured in duplicates.

### Statistical analysis

For analysis of sex differences between groups a Chi-square test was performed, and for analysis of age differences between groups a Kruskal-Wallis test was applied. To compare cell frequencies or numbers between healthy controls, symptomatic controls, untreated and natalizumab-treated patients with RRMS, a Kruskal-Wallis and *post-hoc* Dunn’s test corrected for multiple comparisons was used. For comparison of DICAM^+^ and DICAM^-^ cell populations a Wilcoxon test was used. For comparison of sDICAM secretion in response to LPS stimulation, paired t-tests between stimulated and unstimulated samples were applied. Correlations were assessed by Spearman rank correlation analysis and corrected for multiple comparisons by FDR. Statistical values < 0.05 was considered statistically significant. Statistical analysis was performed using GraphPad Prism 10.1.

## Results

### DICAM is primarily expressed on CD14+ mononuclear phagocytes in CSF

**Figure 1:**
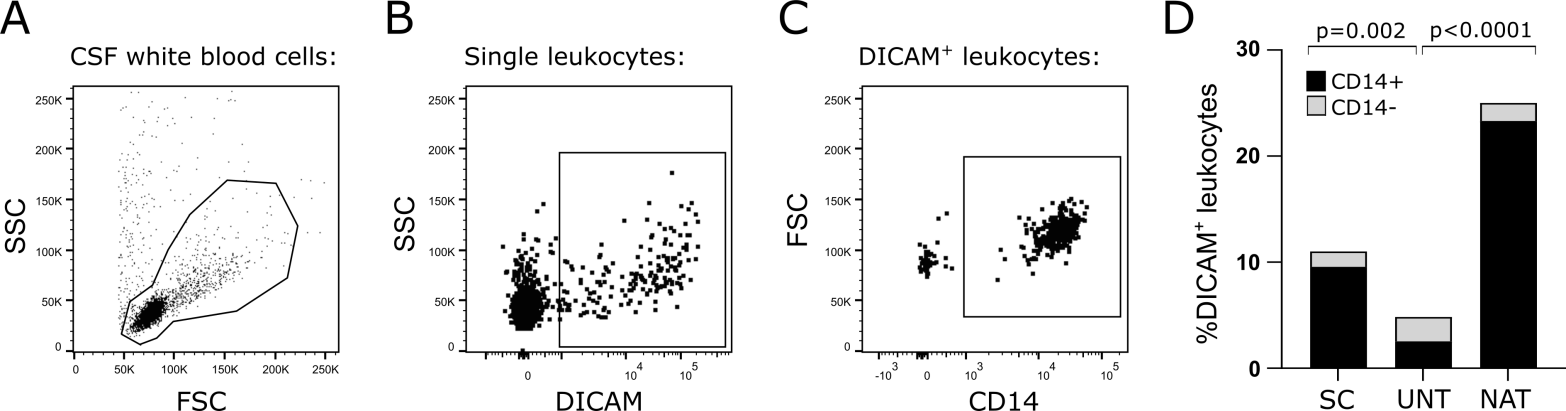
DICAM is primarily expressed on mononuclear phagocytes in CSF. **(A–C)**. Flow cytometry gating strategy. The CSF leukocyte population was defined **(A)**, doublet cells excluded, DICAM^+^ leukocytes gated **(B)**, and CD14^+^ DICAM^+^ cells found **(C)**. **(D)** Frequency of DICAM^+^ leukocytes in CSF of 14 symptomatic controls (SC), 21 untreated patients with RRMS (UNT) and 12 natalizumab-treated patients with RRMS (NAT). Black bars represent percent CD14^+^ DICAM^+^ leukocytes (of total DICAM^+^ leukocytes), grey bars CD14^-^ DICAM^+^ leukocytes (of total DICAM^+^ leukocytes).

To investigate a possible role of DICAM in neuroinflammation, we collected CSF samples from 14 symptomatic controls, 21 treatment-naïve patients with RRMS, and 12 patients treated with natalizumab, and used flow cytometry to determine the frequency of DICAM^+^ leukocytes (**Figures 1A–C**). This showed that 11% (7.3; 21) of CSF leukocytes expressed DICAM in controls, 4.6% (2.9; 6.2) in untreated patients, and 25% (17; 30) in patients treated with natalizumab; median (IQR). Compared to both symptomatic controls (p=0.002) and natalizumab-treated patients (p<0.0001), untreated patients had a significantly lower frequency of DICAM^+^ cells (**Figure 1D**). Furthermore, the majority of DICAM^+^ leukocytes in both controls (87%) and natalizumab-treated patients (93%) were CD14^+^ mononuclear phagocytes (**Figure 1D**). Of the few DICAM^+^ cells in CSF of treatment-naïve patients, 55% were CD14^+^ mononuclear phagocytes (**Figure 1D**). CD14^+^ mononuclear phagocytes are hereafter referred to as mononuclear phagocytes.

### Low prevalence of intrathecal DICAM+ mononuclear phagocytes in untreated patients

**Figure 2:**
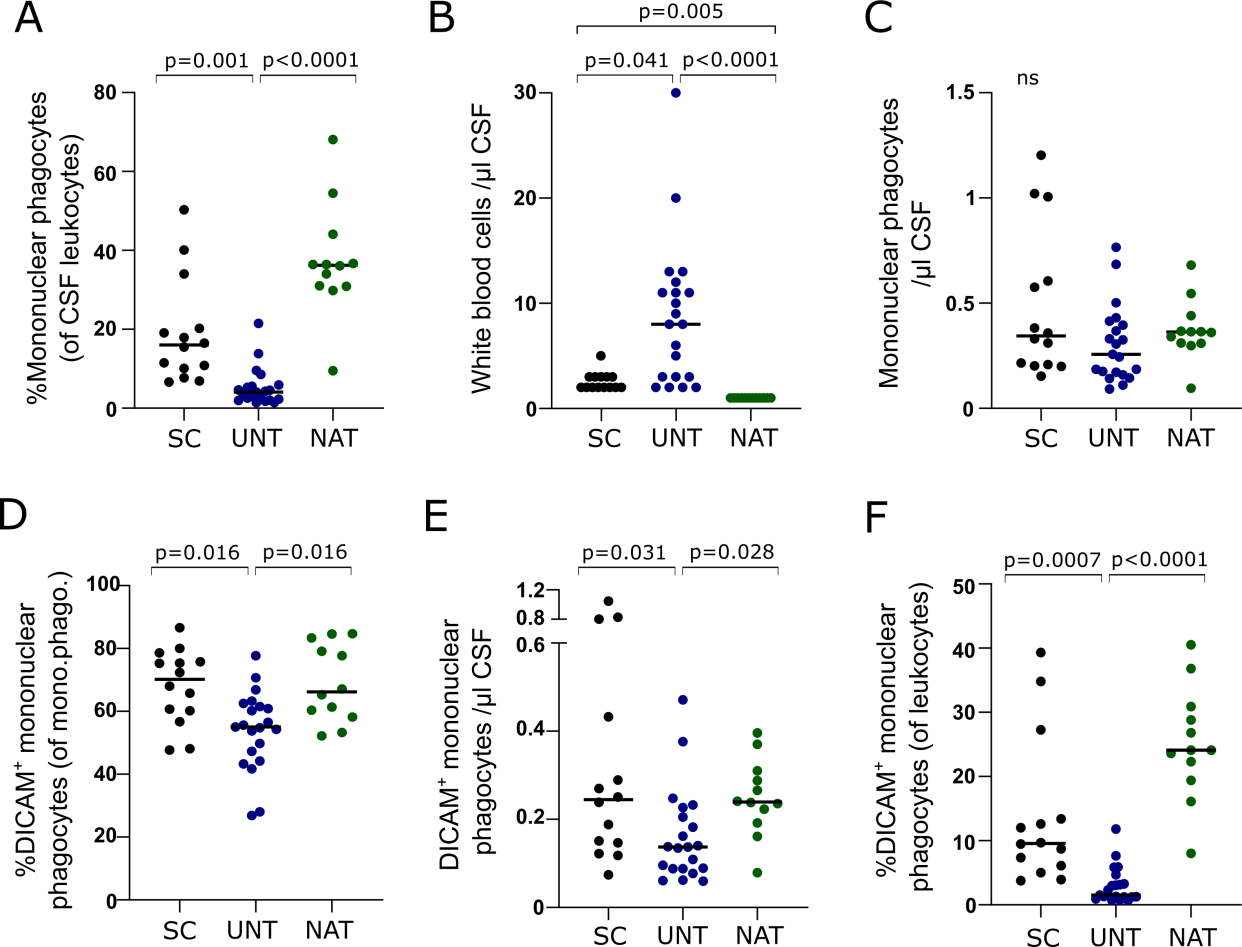
Low prevalence of intrathecal DICAM^+^ mononuclear phagocytes in untreated patients. **(A)** Frequency of mononuclear phagocytes in CSF of 14 symptomatic controls (SC), 21 untreated patients with RRMS (UNT) and 12 natalizumab-treated patients with RRMS (NAT). **(B)** White blood cell count in CSF. **(C)** Cell count of mononuclear phagocytes in CSF. **(D–F)**. Frequency and absolute number of DICAM^+^ mononuclear phagocytes in CSF. Median value is shown for all groups analyzed.

Further analyzing mononuclear phagocytes in the CSF showed that the frequency of mononuclear phagocytes among leukocytes was greatly reduced in untreated patients compared to symptomatic controls (p=0.001) and natalizumab-treated patients (p<0.0001); **Figure 2A**. Most untreated patients had CSF pleocytosis (**Figure 2B**) and we observed that the absolute number of mononuclear phagocytes in untreated patients was similar to both symptomatic controls and natalizumab-treated patients (**Figure 2C**).

Also, the frequency of mononuclear phagocytes expressing DICAM was lower in untreated patients compared to both symptomatic controls (p=0.016) and natalizumab-treated patients (p=0.016); **Figure 2D**. Despite an equal number of intrathecal mononuclear phagocytes in the three groups, the absolute number of DICAM^+^ mononuclear phagocytes in untreated patients were lower compared to both symptomatic controls (p=0.031) and natalizumab-treated patients (p=0.028); **Figure 2E**. This difference was further emphasized by the observation that DICAM^+^ mononuclear phagocytes constituted 24% of all leukocytes in CSF of natalizumab-treated patients and 10% in controls, compared to only 1.5% in untreated patients (**Figure 2F**).

### Surface phenotype of intrathecal DICAM+ mononuclear phagocytes

**Figure 3:**
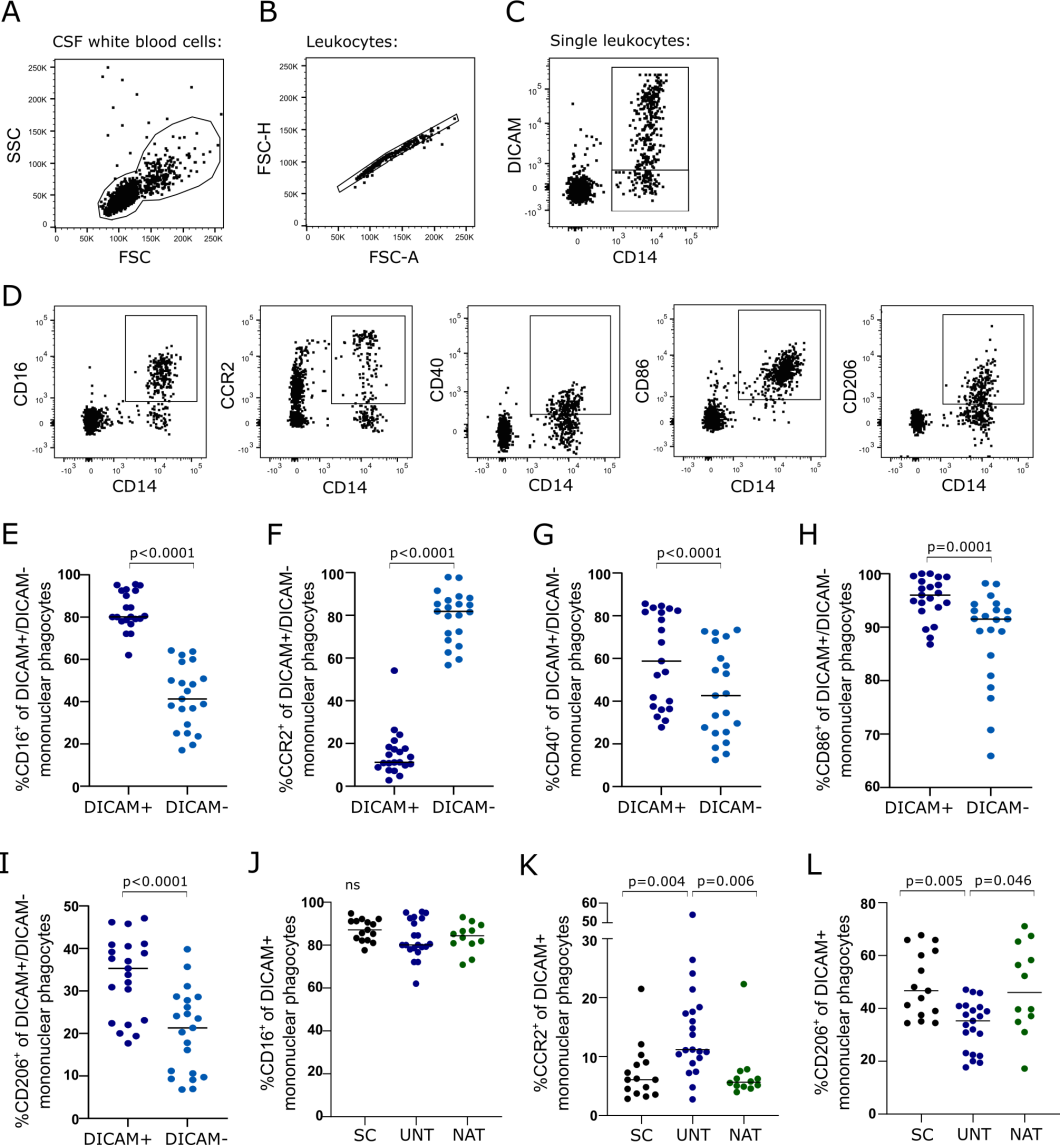
Surface phenotype of intrathecal DICAM^+^ mononuclear phagocytes. **(A–C)**. Flow cytometry gating strategy. The CSF leukocyte population was defined **(A)**, doublet cells excluded **(B)**, CD14^+^DICAM^-^ and CD14^+^DICAM^+^ mononuclear phagocytes defined **(C)**. **(D)** Mononuclear phagocytes expressing the surface markers CD16, CCR2, CD40, CD86, and CD206 were hereafter defined. **(E–I).** Frequency of CD16^+^ **(E)**, CCR2^+^ **(F)**, CD40^+^ **(G)**, CD86^+^ **(H)**, and CD206^+^ **(I)** DICAM^+^ and DICAM^-^ mononuclear phagocytes in CSF of 21 untreated patients with RRMS. **(J–L)**. Frequency of CD16^+^ **(J)**, CCR2^+^ **(K)**, and CD206^+^ **(L)** DICAM^+^ mononuclear phagocytes of 14 symptomatic controls (Control), 21 untreated patients with RRMS (UNT) and 12 natalizumab-treated patients with RRMS (NAT). Median value is shown for all groups analyzed.

To understand the role of intrathecal DICAM^+^ mononuclear phagocytes in neuroinflammation, we used flow cytometry to characterize the surface expression of various proteins associated with the function of mononuclear phagocytes in the CSF of untreated patients with RRMS. For this, the following gating strategi was applied: the CSF leukocyte population was defined (**Figure 3A**), doublet cells excluded (**Figure 3B**), and CD14^+^DICAM^-^ and CD14^+^DICAM^+^ mononuclear phagocytes defined (**Figure 3C**). Expression of the surface markers CD16, CCR2, CD40, CD86, and CD206 where hereafter defined (**Figure 3D**) and the gates applied to both the DICAM^+^ and DICAM^-^ mononuclear phagocyte population. This analysis showed that DICAM^+^ mononuclear phagocytes in CSF predominantly were CD16^+^ and CCR2^-^ in contrast to DICAM^-^ mononuclear phagocytes (p<0.0001); **Figures 3E, F**. Additionally, DICAM^+^ mononuclear phagocytes had an increased expression of CD40 (p<0.0001), CD86 (p=0.0001), and CD206 (p<0.0001) compared to DICAM^-^ mononuclear phagocytes, **Figures 3G–I**. Also, like DICAM^-^ mononuclear phagocytes, 100% of DICAM^+^ mononuclear phagocytes expressed CD49d, which is one of the two subunits of the adhesion molecule Very Late Activation Antigen-4 (VLA-4; data not shown). The same was found in symptomatic controls and natalizumab-treated patients (**Supplementary Figure 1**).

Comparing the phenotype of DICAM^+^ mononuclear phagocytes of untreated patients with symptomatic controls and natalizumab-treated patients showed a similar frequency of intrathecal DICAM^+^ mononuclear phagocytes expressing CD16, CD40, CD86 and CD49d between the three groups (**Figure 3J**, and data not shown). In contrast, the frequency of DICAM^+^ mononuclear phagocytes expressing CCR2 in untreated patients compared to controls and natalizumab-treated patients was increased (p=0.004 and 0.006) and the expression of CD206 decreased (p=0.005 and 0.046) (**Figures 3K, L**).

### Few circulating monocytes express DICAM

**Figure 4:**
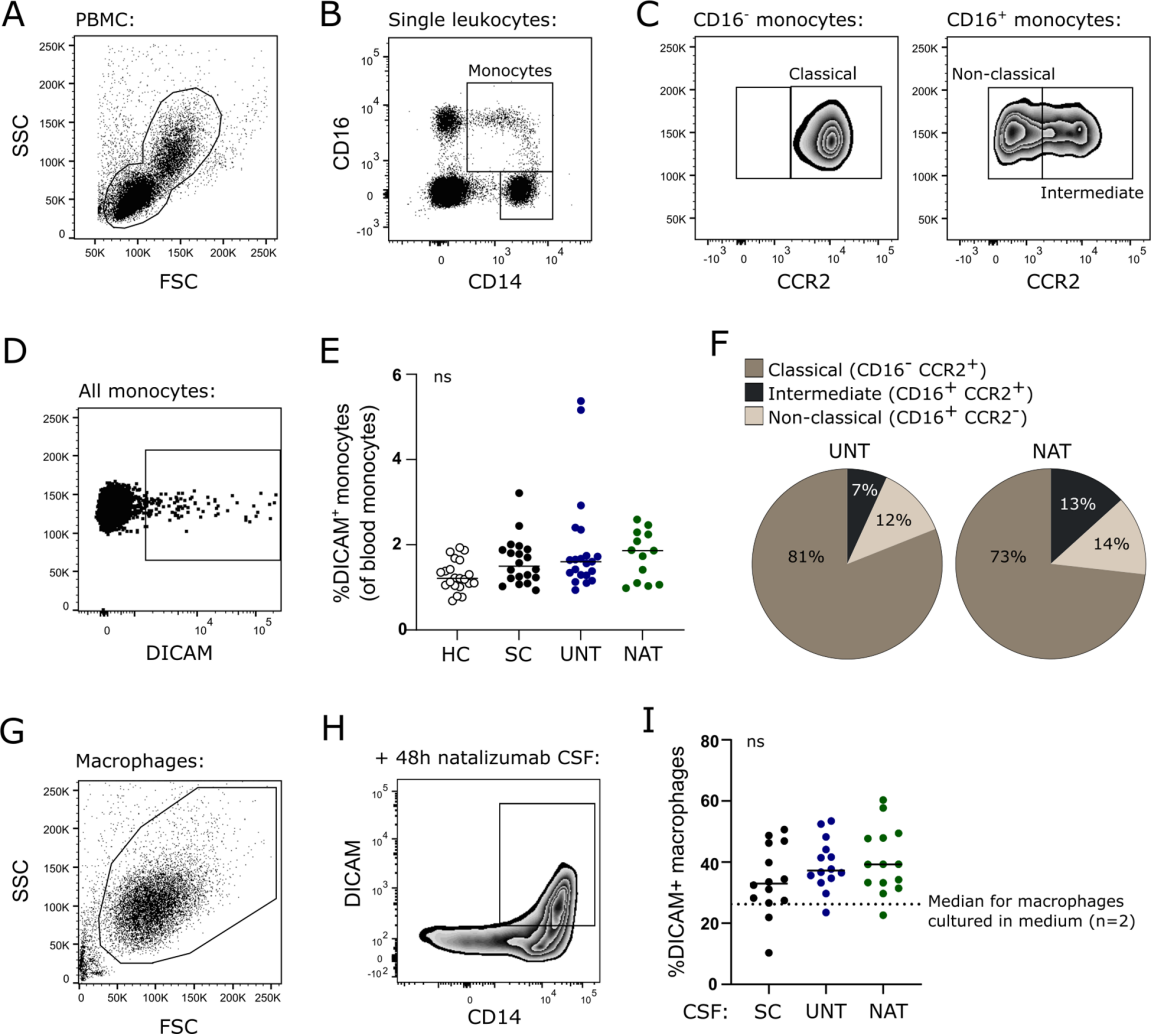
DICAM is upregulated following monocyte differentiation to macrophages. **(A–D)**. Flow cytometry gating strategy. The blood leukocyte population was defined **(A)**, doublet cells excluded, CD14^+^CD16^-^ and CD14^+^CD16^+^ monocyte populations gated **(B)**, and classical CD16^-^ CCR2^+^, CD16^+^ CCR2^+^ intermediate, and CD16^+^ CCR2^-^ non-classical monocyte subpopulations defined **(C)**, and percent DICAM^+^ monocytes found **(D)**. **(E)** Frequency of DICAM^+^ leukocytes in blood of 21 healthy controls (HC), 21 symptomatic controls (SC), 21 untreated patients with RRMS (UNT) and 13 natalizumab-treated patients with RRMS (NAT). **(F)** Distribution of circulating DICAM^+^ monocyte subsets of untreated and natalizumab-treated patients. **(G)** Flow cytometry dot plot example showing *in vitro* differentiated macrophages. **(H)** DICAM^+^ macrophages cultured in CSF from natalizumab-treated patients with RRMS. Plot shown are after doublet cell and dead cell exclusion. **(I)** Frequency of DICAM^+^ macrophages (DICAM^+^ macrophages of total macrophages) after 48 h culture in CSF from 14 symptomatic controls (SC), 14 untreated patients with RRMS (UNT) and 14 natalizumab-treated patients with RRMS (NAT). Dotted line shows median frequency of macrophages grown for 48 h in medium with no CSF (n=2). Median value is shown for all groups analyzed.

In the blood, DICAM is expressed on less than 2% of circulating CD14^+^ monocytes, with no difference between healthy controls (n=21), symptomatic controls (n=21), untreated (n=21) or natalizumab-treated (n=13) patients with RRMS (**Figures 4D, E**). Subdividing circulating DICAM^+^ monocytes into classical (CD16^-^), intermediate (CD16^+^CCR2^+^) and non-classical (CD16^+^CCR2^-^) monocytes (**Figures 4A–C**) showed a higher frequency of DICAM^+^ intermediate monocytes in natalizumab-treated patients compared to both symptomatic controls (p=0.029) and untreated patients (p=0.035), **Figure 4F**.

### DICAM is strongly upregulated following monocyte differentiation to macrophages

To investigate if the enrichment of DICAM^+^ mononuclear phagocytes observed in the CSF was due to selective recruitment of DICAM^+^ monocytes from the blood, differentiation of recruited monocytes to CNS border-patrolling macrophages or an effect of the local intrathecal microenvironment, monocytes from healthy controls were purified and cultured under macrophage differentiating conditions for 6 days. Thereafter, macrophages were grown in medium or CSF from either symptomatic control subjects (n=14), untreated patients (n=14), or natalizumab-treated patients (n=14) for an additional 2 days and DICAM expression was measured (**Figures 4G, H**). Following macrophage differentiation, we observed a strong increase in DICAM^+^ cells to a mean of 26% (dotted line in **Figure 4I**). This increase was marginally elevated when cells were cultured in CSF; however, not significantly and all CSF-samples affected the expression of DICAM equally (**Figure 4I**). The experiment was repeated, and the same observations obtained (data not shown).

### Soluble DICAM in CSF is increased in natalizumab-treated patients and correlate negatively with inflammation biomarkers

**Figure 5:**
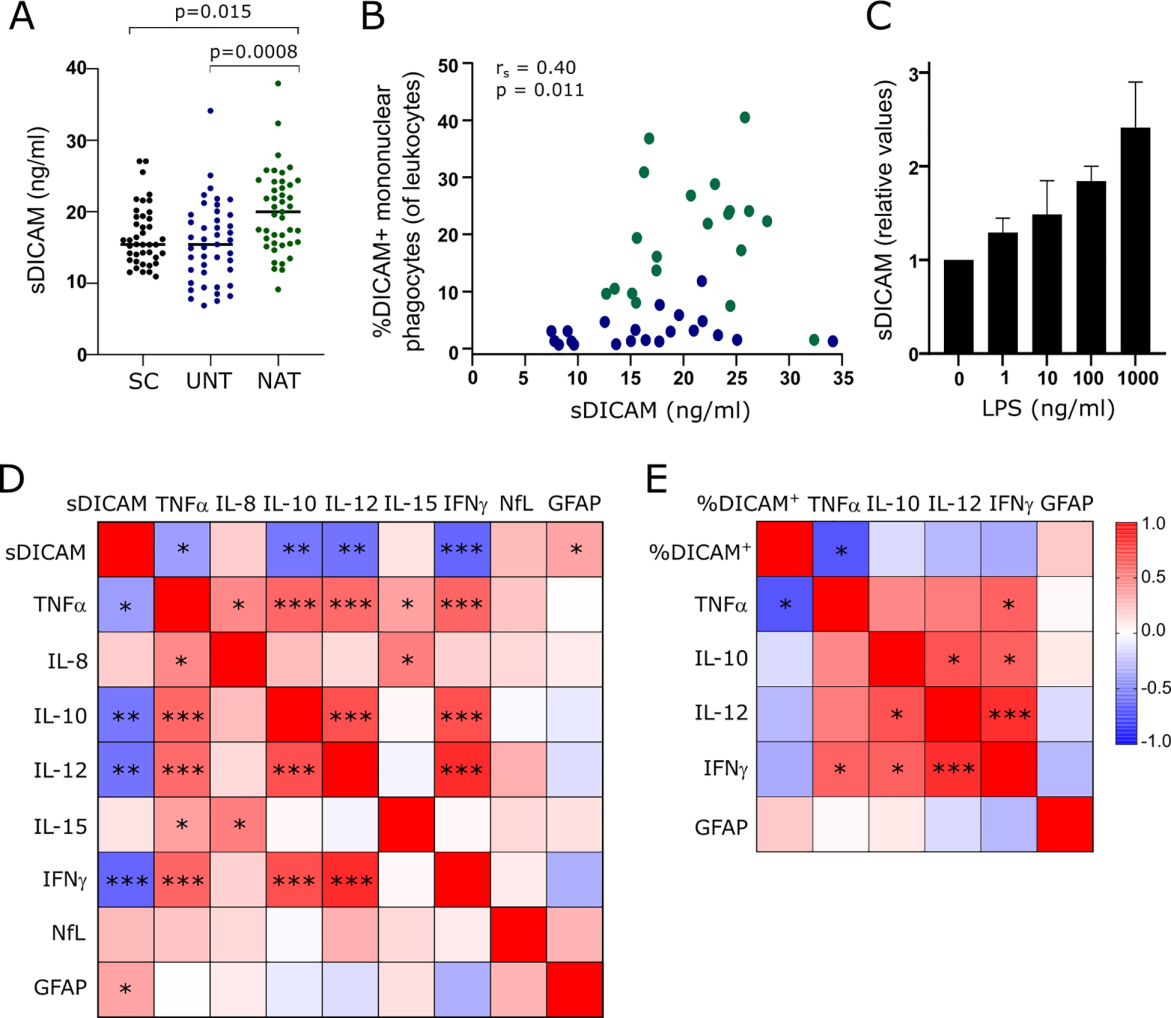
sDICAM, DICAM^+^ mononuclear phagocytes and biomarkers in CSF. **(A)** sDICAM concentration in CSF of 41 symptomatic controls (SC), 43 untreated patients with RRMS (UNT) and 42 natalizumab-treated patients with RRMS (NAT). Median value is shown for all groups analyzed. **(B)** Correlation analysis between sDICAM and percent DICAM^+^ mononuclear phagocytes in CSF of patients with RRMS (blue circles represents untreated patients, green represents natalizumab treated patients; r_s_ is the spearman correlation coefficient). **(C)** sDICAM secreted by mononuclear phagocytes of three healthy donors stimulated for 14 h with the indicated concentrations of LPS. Mean+SD shown. **(D)** Correlation matrix of sDICAM and biomarkers in CSF of 43 untreated patients with RRMS. **(E)** Correlation matrix of percent DICAM^+^ mononuclear phagocytes and biomarkers in CSF of 18 untreated patients with RRMS. Only biomarkers with a significant correlation to sDICAM was included in the analysis of percent DICAM^+^ mononuclear phagocytes (of CSF leukocytes). Correlation coefficients are depicted by colors. FDR-corrected p-values (q-values) are depicted. * q<0.05; **q<0.005; ***q<0.0005.

In addition to a membrane bound form, DICAM exists in a soluble form (sDICAM). To investigate if sDICAM is present in CSF and whether there is a difference between patients and symptomatic controls, we measured sDICAM in CSF from 41 symptomatic controls, 43 treatment-naïve and 42 natalizumab-treated patients by ELISA. sDICAM could be detected in all CSF samples analyzed. The analysis showed higher sDICAM concentrations in natalizumab-treated patients compared to both untreated patients (p=0.0008) and symptomatic controls (p=0.015), **Figure 5A**. The variation in duration of natalizumab treatment and time since last treatment between treated patients may influence the level of sDICAM in CSF. To analyze this, we performed a correlation analysis between these parameters and the concentration of sDICAM and found no correlation (p=0.084, r_s_=0.27 and p=0.124, r_s_=-0.24; respectively).

Besides mononuclear phagocytes, a small population of T cells (6), endothelial cells (9), and astrocytes (12) have been shown to express DICAM. The measured sDICAM in CSF is therefore likely of mixed origin; however, we found a positive correlation between sDICAM and the frequency of DICAM^+^ mononuclear phagocytes in CSF of patients with RRMS (p=0.011, r_s_=0.40; **Figure 5B**). Also, we found that mononuclear phagocytes stimulated *in vitro* with LPS for 14 h secreted sDICAM (**Figure 5C**); altogether suggesting that these cells are major contributors to CSF sDICAM.

To investigate a clinical implication of DICAM in MS, we analyzed a possible association between sDICAM in CSF of 43 untreated patients with RRMS and the biomarker of neuroaxonal damage neurofilament light chain (NfL) (17) and the astrocyte activity biomarker glial fibrillary acidic protein (GFAP) (18). This showed a weak association between sDICAM and an increased GFAP (q=0.04; r_s_=0.35); **Figure 5D**. Further analyzing an association between sDICAM in CSF and various cytokines showed a negative correlation between sDICAM and TNFα (q=0.028; r_s_=-0.39), IFNγ (q=0.0002; r_s_=-0.60), IL-12 (q=0.0008; r_s_=-0.55), and IL-10 (q=0.001; r_s_=0.54); **Figure 5D**. To improve a link between these observations and the presence of DICAM^+^ mononuclear phagocytes in CSF, we next analyzed correlations between the frequency of DICAM^+^ mononuclear phagocytes in untreated patients with RRMS and the biomarkers that correlated with sDICAM. Despite a considerably lower sample size in this analysis (n=18), we found a negative correlation between DICAM^+^ mononuclear phagocytes and TNFα (q=0.009; r_s_=-0.66); **Figure 5E**. All statistical values shown are FDR-corrected p-values (q-values).

### DICAM attenuates LPS-induced production of TNF in mononuclear phagocytes

**Figure 6:**
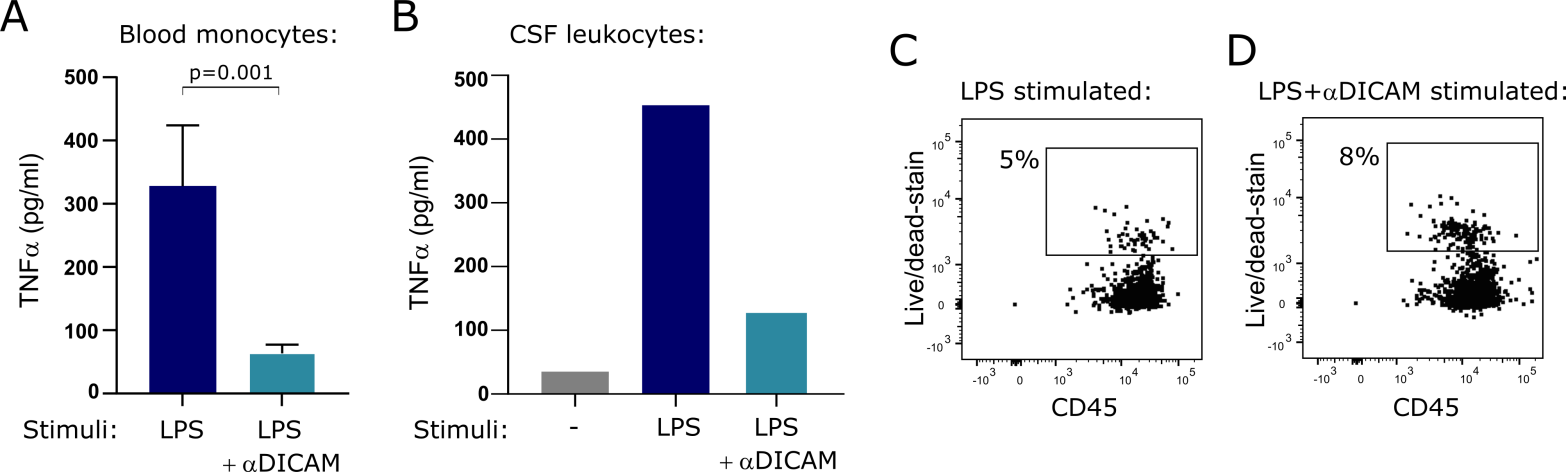
DICAM attenuates production of TNFα. **(A)**. TNFa production by monocytes from 7 healthy donors after 6 h of stimulation with 10 ng/ml LPS with or without DICAM triggering by anti-DICAM antibodies. Monocytes stimulated by LPS are referred to as LPS and monocytes stimulated by LPS and anti-DICAM antibody are referred to as LPS+aDICAM on the graph. Mean+SD shown. **(B)**. TNFa production by mononuclear phagocytes in CSF after 6 h of stimulation with 10 ng/ml LPS with or without anti-DICAM antibodies. **(C, D)** Flow cytometry dot plot example of cell death analysis of LPS-stimulated monocytes from the healthy donors.

Studies have indicated a role of DICAM in suppressing intracellular signaling pathways (9) and lipopolysaccharide (LPS)-induced production of proinflammatory cytokines including TNFα (10). Based on our data suggesting a relation between DICAM and TNFα in CSF, we investigated DICAM’s direct ability to influence TNFα-production in mononuclear phagocytes. To study this functional property of DICAM, we sorted monocytes into CD14^+^DICAM^-^ and CD14^+^DICAM^+^ cells using antibodies targeting CD14 and DICAM, at a low temperature to ensure that no signals were induced through the DICAM-receptor by the anti-DICAM antibody during sorting. The two sorted populations were hereafter cultured in 10 ng/ml LPS for 6 h at 37°C allowing anti-DICAM induced signaling through surface expressed DICAM. The culture supernatants were collected, and TNFα-concentration measured. This showed a prompt LPS-induced production of TNFα without triggering of DICAM (**Figure 6A**, LPS); a production that was strongly reduced by anti-DICAM antibody triggering of DICAM (**Figure 6A**; LPS+αDICAM, p=0.001). The experiment was repeated three times with a total of 7 donors. To validate this finding, the same cocktail of antibodies and LPS was added to freshly drawn CSF-cells from a patient with RRMS, and TNFα production was measured. Likewise, this showed that a signal through DICAM diminished TNFα production (**Figure 6B**). As a previous study described that DICAM accelerated apoptosis in endothelial cells (9) we measured cell death after 6h LPS-stimulation in the two sorted populations to ensure that the decrease in TNFα-production observed was not simply a result of cell death. This showed that cells in which DICAM was triggered were more sensitive to apoptosis, but not to a large degree (**Figures 6C, D**). Altogether, these data indicate that a functional property of DICAM is to attenuate LPS-induced TNFα production in mononuclear phagocytes.

## Discussion

With this study we investigated a possible role of DICAM in controlling neuroinflammation. Our findings showed that the primary CSF-resident immune cell to express DICAM was mononuclear phagocytes, including CSF-infiltrating monocytes and differentiated CNS-border patrolling macrophages. Investigating symptomatic controls as a representative of steady-state conditions showed a high prevalence of DICAM^+^ mononuclear phagocytes, both in frequency and numbers, compared to treatment-naïve patients with MS. When patients were treated with natalizumab, an antibody blocking migration of VLA-4^+^ blood leukocytes to the CNS (19), we observed that DICAM^+^ monocytes were still recruited to the CSF despite VLA-4 expression and that the frequency and numbers in CSF resembled those observed in the symptomatic controls. The observed decrease in DICAM^+^ mononuclear phagocytes in untreated patients compared to both controls and natalizumab-treated individuals with no disease activity may indicate a regulatory role of this cell subset. The idea of DICAM as a mechanism to control inflammation is in line with previous studies showing that DICAM has a protective role in the mouse model of experimental autoimmune optic neuritis (13) and experimental colitis (11), that DICAM can protect against renal tubular injury through its anti-inflammatory properties (10), and that DICAM is involved in cancer-related signaling pathways promoting tumor malignancy and tumor immune dysfunction (20). Furthermore, a study described how DICAM secreted in extracellular vesicles during neuroinflammation had the potential to control the activity of microglia, a mononuclear phagocyte of CNS origin, and overall neuroinflammation (12).

The mode of DICAM to control inflammation has not been thoroughly investigated; however, signals through surface-expressed DICAM likely suppress activation-induced intracellular signaling events of the AKT and p38 MAP kinase signaling pathways (9). DICAM-triggering reduces production of both integrins and proinflammatory cytokines including TNFα, IL-1β, and IL-6 (10), and increases the level of occludin (10) and stabilizes barrier function (11). In coherence, we found that triggering of surface expressed DICAM on mononuclear phagocytes during LPS-stimulation effectively reduced the production of TNFα.

DICAM has been shown to interact homophilic with DICAM expressed on the same cell and heterophilic with αVβ3 (7, 21), but other ligands likely exist. If sDICAM can engage with DICAM on adjacent cells, our observation of sDICAM in CSF and the ability of mononuclear phagocytes to secrete DICAM, along with a study documenting astrocyte secretion of DICAM in extracellular vesicles (12) may imply a regulatory role of DICAM distinct from the site where it was produced. In support of this notion, we observed an increased level of sDICAM in CSF of natalizumab-treated patients, possibly contributing to the attenuation of inflammation observed in these patients; and furthermore, we found a negative correlation between the concentration of sDICAM and TNFα, IFNγ, IL-12, and IL-10 in CSF of patients with MS. This finding was substantiated by a negative correlation between DICAM^+^ mononuclear phagocytes and TNFα in CSF. Also, we observed a tendency to a negative correlation with IFNγ and IL-12; however, likely due to lack of power (n=18 versus n=43 in the sDICAM analysis) this was non-significant.

In the blood, only few monocytes expressing DICAM were found (<2% versus approximately 70% in CSF). We furthermore observed that DICAM was strongly upregulated *in vitro* following differentiation of monocytes to macrophages; suggesting DICAM to be a macrophage marker. It is therefore possible that circulating DICAM^+^ mononuclear phagocytes represent a small population of macrophages rather than undifferentiated monocytes. DICAM has previously been suggested to be an adhesion molecule on a small subtype of inflammatory T cells, Th17 cells, involved in CNS-migration independent of the otherwise mandatory VLA-4 (6). According to this and our observation that mononuclear phagocytes are still able to transmigrate to CNS despite natalizumab treatment, indicates that selective recruitment of the peripheral pool of DICAM-expressing mononuclear phagocytes to the CNS is possible, although the majority of intrathecal DICAM^+^ mononuclear phagocytes likely are generated upon differentiation from infiltrated monocytes to CNS border-patrolling macrophages.

Depending on the cytokine milieu in CNS, infiltrating monocytes are capable of adopting either a pro- or anti-inflammatory macrophage phenotype, a phenomenon of key relevance in MS where the pro-inflammatory state dominates (22). As we observed a decreased prevalence of DICAM^+^ mononuclear phagocytes in CSF of untreated patients with MS, we investigated the impact of the CSF-environment on DICAM expression. For this, we cultured *in vitro* differentiated macrophages in cell-free CSF from either symptomatic control subjects, untreated or natalizumab-treated patients and found no significant change in DICAM surface-expression following CSF incubation, and also not between the three groups. The local CNS-environment mirrored by cell-free CSF may therefore not be the key factor of DICAM upregulation in differentiating macrophages.

Phenotypically, intrathecal DICAM^+^ mononuclear phagocytes are predominantly CD16^+^ and CCR2^-^. Additionally, a high frequency of DICAM^+^ mononuclear phagocytes in CSF express CD40, CD86 and CD206. Where CD206 is considered representative of macrophage polarization towards an anti-inflammatory and homeostatic phenotype, CD40 and CD86 are associated with a pro-inflammatory state where TNFα, IFNγ, and IL-12 are induced and inflammatory Th cells are primed (23). However, we observe a negative correlation between these three cytokines and DICAM^+^ mononuclear phagocytes; one could therefore speculate that DICAM^+^ mononuclear phagocytes may use CD40 and CD86 to interact with inflammatory T cells to control their activity through DICAM-induced signals in the target cell. This theory; however, needs to be investigated in future studies. Whereas 96-99% of all DICAM^+^ CFS mononuclear cells from both symptomatic controls, untreated and natalizumab-treated patients expressed CD86, significant less DICAM^+^ CFS mononuclear cells from untreated patients (35%) compared to both symptomatic controls (47%) and natalizumab-treated patients (46%) expressed CD206. This observed difference in phenotype may be due to plasticity of mononuclear phagocytes likely modelled by the intrathecal environment. This change in DICAM^+^ cell phenotype may finetune their function and hence their capacity to adapt an anti-inflammatory response. Unfortunately, we did not include these phenotype markers in our *in vitro* experiment investigating the impact of CFS-environment on the polarization of mononuclear phagocytes.

A previous study showed that the main T cell population to express DICAM was Th17 cells (6). As observed with monocytes inducing DICAM expression upon differentiation to macrophages in the current study, DICAM induction was observed when CD4^+^ T cells were differentiated to Th17 cells *in vitro* (6). Th17 cells are considered proinflammatory and implicated in the MS pathogenesis. Blocking DICAM reduced the migration of Th17 cells into the CNS and led to reduced disease severity in the animal model experimental autoimmune encephalomyelitis (EAE) (6). This identifies DICAM as a CNS-migration molecule for Th17 cells and confirms the importance of Th17 cells for neuroinflammation. In this study we propose that DICAM expression on mononuclear phagocytes is a way to control the activity of these cells, and similarly, DICAM-expression on Th17 cells may also control the activity of Th17 cells if they enter the CNS. However, an anti-inflammatory role of DICAM on Th17 cells awaits further investigations.

Altogether, our study indicates a protective immunomodulatory role of DICAM^+^ mononuclear phagocytes in neuroinflammation in multiple sclerosis and proposes that the regulatory effect is directly delivered by DICAM: triggering of surface expressed DICAM on mononuclear phagocytes (or other cells expressing DICAM) effectively reduce their production of pro-inflammatory cytokines. If sDICAM can engage with surface expressed DICAM on adjacent cells through a homophilic-interaction, sDICAM may additionally regulate inflammation in a paracrine way. The observed relationship between CSF concentrations of sDICAM and lower concentrations of pro-inflammatory cytokines suggests that sDICAM might be useful as a prognostic biomarker in newly diagnosed patients with MS. This should be addressed in future, prospective studies. It is also possible that treatment with agonistic anti-DICAM antibodies might be useful for suppressing pro-inflammatory signaling in mononuclear phagocytes.

## Data Availability

The original contributions presented in the study are included in the article/**Supplementary Material**. Further inquiries can be directed to the corresponding author/s.
