# Supplementary figures and images for: A role for DICAM+ mononuclear phagocytes in controlling neuroinflammation in multiple sclerosis
Source: Front Immunol. 2025 Jul 28;16:1628398. doi: 10.3389/fimmu.2025.1628398 (PMC12336262; doi:10.3389/fimmu.2025.1628398)

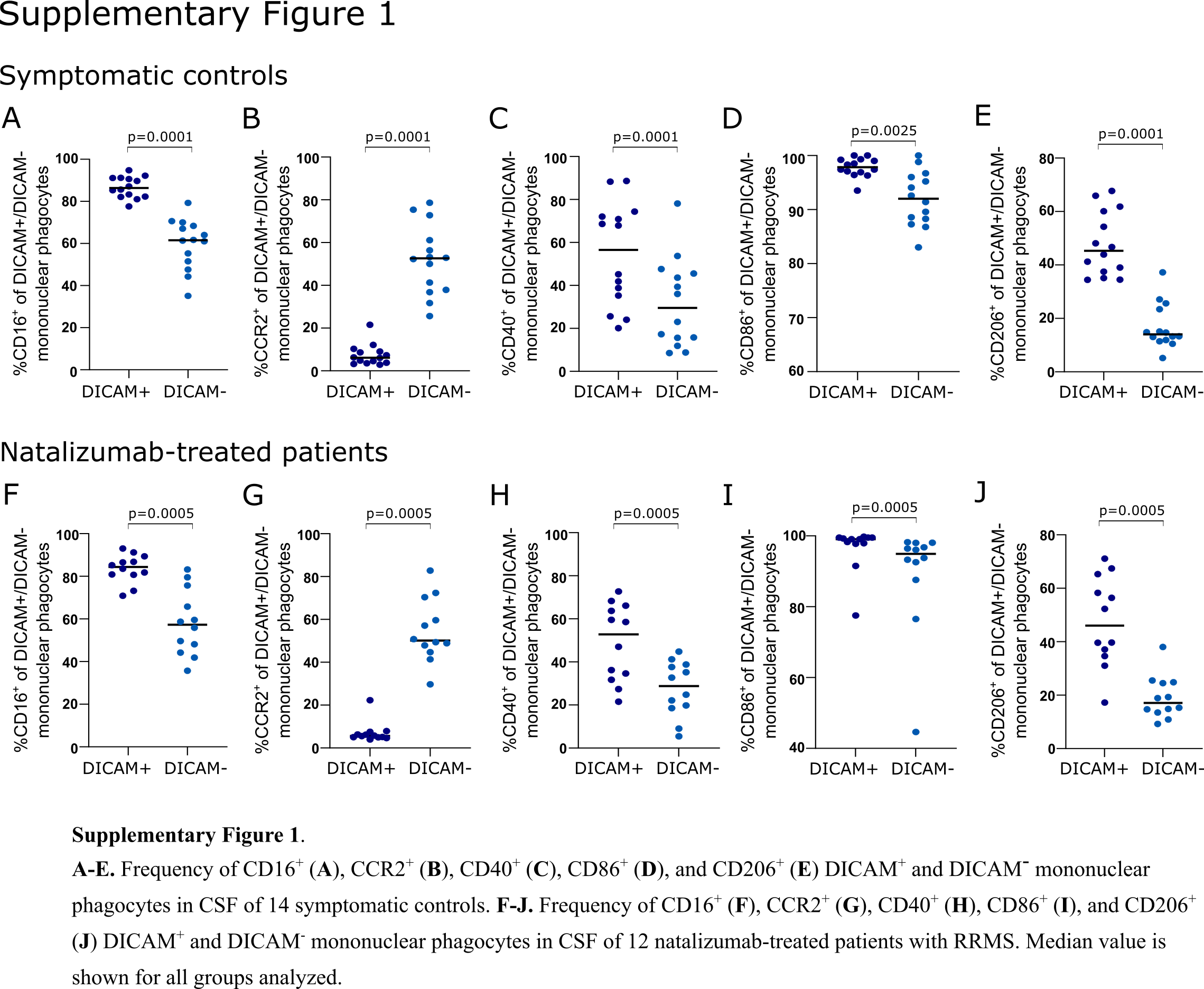

Supplement: Supplementary file 1 [file Image1.tiff]
